# Editorial: Stillbirths in low-middle income countries: challenges & experiences

**DOI:** 10.3389/fgwh.2023.1240004

**Published:** 2023-07-25

**Authors:** Bethany Atkins, Dimitrios Siassakos, Neelam Aggarwal

**Affiliations:** ^1^ University College London, London, United Kingdom; ^2^ Department of Obstetrics and Gynecology, Post Graduate Institute of Medical Education and Research (PGIMER), Chandigarh, India

**Keywords:** stillbirth, perinatal death, global health, low and mid income countries, stillbirth (SB), prevention, pregnancy loss

**Editorial on the Research Topic** Stillbirths in low-middle income countries: challenges & experiences

Almost half of the 2 million stillbirths that occur globally each year are thought to be preventable (1). The burden of stillbirths falls predominantly on low- and middle-income countries, where 84% occur. The collection of papers in this Research Topic highlights potential interventions to end preventable stillbirths.

Prevention of stillbirth requires understanding of both direct and indirect causes, as well as the health systems and wider contexts within which women and birthing people deliver their babies. Improving skilled birth attendance, for example, without equipping families first with the necessary tools to plan pregnancies and seek pre-conception care, will have only a partial impact.

Efforts to reduce stillbirth incidence are often hampered by poor recording of when stillbirths are occurring and why, an issue clearly highlighted by Milton et al. in their observational study of stillbirth determinants in Kano, Nigeria. Their study found surprisingly mixed data between the two facilities surveyed; higher household income for example was associated with increased likelihood of stillbirth in one facility, and reduced likelihood in the other. The poorly understood causality of stillbirth in specific contexts is further highlighted by Swarray-Deen et al. observational study of healthcare workers’ awareness of perinatal autopsy in Ethiopia. They demonstrate a low uptake of freely-available postmortem investigation, associated with lack of training and awareness among healthcare professionals. Investigation of stillbirths within resources is a key principle (RESPECT) of care (2), and may help to reduce the stigma attached to its occurrence in some settings (3).

Globally, around 40% of stillbirths occur intrapartum (1). In Europe, North America, Australia and New Zealand, only 6% of stillbirths occur during labour but this figure is higher in all other regions, increasing up to 49% in sub-Saharan Africa (1). Most stillbirths during labour are thought to be preventable with high-quality intrapartum care, including early detection of complications and escalation of care as appropriate. The low-cost sensor glove pioneered by Jaufuraully et al. could address one important cause of stillbirth; undetected obstructed labour. The glove contains a sensor on the tip of the index finger, which facilitates identification of the fetal cranial sutures, and therefore the position of the anterior and posterior fontanelles of the fetal skull. While it is currently in the early stages of development, this device has the potential to train birth attendants, upskilling them with minimal additional time demands, essential in many health systems with low proportions of healthcare workers per capita. In a pilot study (Jaufuraully personal communication), medical students achieved very high levels of accuracy in diagnosing malposition with the glove.

Beyond the immediate period of pregnancy and loss, pre-conception and inter-conception advice is often neglected when addressing stillbirth, despite being a key principle of care (RESPECT—(2)). Baynes et al. illustrate the complexity of improving family planning provision in low- and middle-income countries, and suggest how implementation science can be used to address some of these barriers.

Underlying all of these improvements is a need for greater training of healthcare professionals. All studies within this series echo the same call; efforts to reduce stillbirths must be targeted and informed by locally-relevant evidence. In addition to stillbirth prevention, future research is also required to expand the provision of bereavement care that is exemplary, supportive, compassionate, and relevant to local settings.
